# Progress in Medicine

**Published:** 1899-09-16

**Authors:** 


### PROGRESS IN MEDICINE.

### PÆDIATRICS.

**Kernig's Sign in Meningitis.**—Attention was directed anew to this sign in the Cavendish Lecture by Dr. Osler,[1] who thinks it has not attracted sufficient notice in England. He points out that it has long been observed in cases of protracted meningitis that the attitude of the lower limbs is one of flexion, the thighs being drawn up towards the abdomen, and the legs in a state of partial contracture. To test for Kernig's sign the patient should be propped up in bed in the sitting position, when on attempting to extend the leg on the thigh there is contraction of the flexors, which prevents the full straightening of the leg. In the recumbent position, on the other hand, the leg can be fully extended. This sign is stated to be present in all forms of meningitis when the spinal meninges are involved, and is supposed to be due to increased irritability of the lumbar and sacral roots, which are stretched in the process of carrying out the test.

Dr. J. B. Herrick[2] has also investigated the "flexor contracture of Kernig" in 19 cases of meningitis. Nine were epidemic cerebro-spinal meningitis, seven were tuberculous, two were pneumococcal, one was syphilitic, with an acute process superadded. Kernig's sign was present in 17 cases, or 89·4 per cent. He specially notes that in the two cases in which it was absent the examination was made a short time before death, and there was a general laxity of all the muscles present, and the patients were in a comatose condition. Just as retraction and rigidity of the neck muscles may be variable, and before death may disappear entirely, so if examined for only once, or very late in the disease, Kernig's sign may not be elicited. Dr. Herrick examined 100 other individuals, healthy and diseased, for Kernig's sign, and found it present in only two cases.

**Syphilis and Hydrocephalus.**—Two forms of syphilitic hydrocephalus have been described by Dr. D'Astruc.[3] The first of these is due to the arrest of development in the brain, which is dependent on the dystrophic influence of the parental disease. In such cases the ordinary manifestations of syphilis will not necessarily be present, and the cerebral malformations are to be regarded as para-syphilitic lesions. Treatment of this form may be tried, but as the damage done to the brain occurs during fœtal life much benefit cannot be expected. In the second form the lesion has been demonstrated to exist in the ventricular ependyma and the choroid plexus where cell infiltration occurs. This is a true syphilitic hydrocephalus, developing during the first months of life, and dependent on the cerebral localisation of an active hereditary disease. Under the influence of specific treatment much improvement may be expected, and complete cures have been recorded.

**Congenital Dislocation of the Hip.**—At a branch meeting of the British Medical Association, Dr. Harold Stiles[4] showed a patient four years old with this affection, who had been successfully treated by Lorenz's bloodless method. The details are as follows: Firm extension is made on the affected limb in the direction of the long axis of the body, and is kept up for ten minutes or more until the tip of the great trochanter is on a level with that of the opposite side. Then, with the child lying on the floor and an assistant steadying the pelvis, the limb is raised to the vertical position, strongly extended, and then very gradually abducted. In a favourable case the head of the bone can be felt to slip into the acetabulum, but if the limb be replaced in the extended position the dislocation recurs. The limb is, therefore, fixed in the abducted position by means of plaster-of-Paris for a period of from six to nine weeks. For some months more, at intervals of a few weeks, the plaster is changed, the position of flexion and abduction being slightly reduced each time. When the limb has been brought back nearly to the normal position the child may be allowed to get up, but for a time must wear a high shoe on the sound leg, so as to throw the weight of the body on the other, and so press the head of the femur into the acetabulum. In the patient shown the result had been perfect as regards walking power, and complete recovery of all movements in the affected limb.

**Nervous Incontinence of Fæces.**—An example of this rare condition, accompanied by incontinence of urine, in a boy of eight years, is recorded by Dr. Crozier Griffith.[5] The double incontinence had persisted since birth, and for a couple of years he had also had twitching of the face and shoulders. There were usually two or three motions daily, and these were not passed unconsciously, but a sudden desire for relief seized the patient, and the bowels acted at once involuntarily. On physical examination no local cause could be determined. Under treatment by Fowler's solution, and increasing doses of tincture of belladonna, the incontinence of fæces rapidly passed off, but the enuresis was unaffected. Cessation of the drug treatment led to a return of the rectal incontinence.

**Eczema in Infants.**—In an editorial article on this subject in the "Archives of Pediatrics"[6] the opinion is expressed that "it is almost the universal belief of pediatric authorities that eczema in infants and young children is largely of intestinal origin." By this is meant not that any particular food is responsible, but that faulty nutrition of the tissues, due to dietary errors, is present. The special delicacy of the skin in young infants, and a gouty or rheumatic parentage, must also be regarded as factors in the production of eczema. Proceeding on these lines pediatrists—as opposed to dermatologists—have advised constitutional as well as local treatment. In the case of infants at the breast attention must be paid to the diet of the mother, who ought to avoid all malt and alcoholic liquors.

[1] Lancet, June 24, 1899. [2] Amer. Jour. Med. Sci., July, 1899. [3] Therap. Gaz., April 15, 1899, and Clin. Jour., May 17, 1899. [4] Pediatrics, June 1, 1899. [5] Arch. of Pediat., June, 1899. [6] Ibid.

